# Unlocking Potential within Health Systems Using Privacy-Preserving Record Linkage: Exploring Chronic Kidney Disease Outcomes through Linked Data Modelling

**DOI:** 10.1055/s-0042-1757174

**Published:** 2022-09-28

**Authors:** David Lim, Sean Randall, Suzanne Robinson, Elizabeth Thomas, James Williamson, Aron Chakera, Kathryn Napier, Carola Schwan, Justin Manuel, Kim Betts, Chris Kane, James Boyd

**Affiliations:** 1 Curtin School of Population Health, Curtin University, Perth, Western Australia, Australia; 2 Deakin Health Economics, Deakin University, Burwood, Victoria, Australia; 3 Medical School, The University of Western Australia, Perth, Western Australia, Australia; 4 WA Department of Health, Perth, Western Australia, Australia; 5 Renal Unit, Sir Charles Gairdner Hospital, Perth, Western Australia, Australia; 6 Curtin Institute for Computation, Curtin University, Perth, Western Australia, Australia; 7 WA Country Health Service, Perth, Western Australia, Australia; 8 WA Primary Health Alliance, Perth, Western Australia, Australia; 9 La Trobe University, Melbourne, Bundoora, Victoria, Australia

**Keywords:** privacy preserving record, linkage accuracy, chronic kidney disease

## Abstract

**Background** Chronic kidney disease (CKD) is a major global health problem that affects approximately one in 10 adults. Up to 90% of individuals with CKD go undetected until its progression to advanced stages, invariably leading to death in the absence of treatment. The project aims to fill information gaps around the burden of CKD in the Western Australian (WA) population, including incidence, prevalence, rate of progression, and economic cost to the health system.

**Methods** Given the sensitivity of the information involved, the project employed a privacy preserving record linkage methodology to link data from four major pathology providers in WA to hospital records, to establish a CKD registry with continuous medical record for individuals with biochemical specification for CKD. This method uses encrypted personal identifying information in a probability-based linkage framework (Bloom filters) to help mitigate risk while maximizing linkage quality.

**Results** The project developed interoperable technology to create a transparent CKD data catalogue which is linkable to other datasets. This technology has been designed to support the aspirations of the research program to provide linked de-identified pathology, morbidity, and mortality data that can be used to derive insights to enable better CKD patient outcomes. The cohort includes over 1 million individuals with creatinine results over the period 2002 to 2021.

**Conclusion** Using linked data from across the care continuum, researchers are able to evaluate the effectiveness of service delivery and provide evidence for policy and program development. The CKD registry will enable an innovative review of the epidemiology of CKD in WA. Linking pathology records can identify cases of CKD that are missed in the early stages due to disaggregation of results, enabling identification of at-risk populations that represent targets for early intervention and management.

## Background and Significance

Chronic kidney disease (CKD) is projected to be the fifth leading cause of death worldwide by the year 2040, 1 with an annual estimate of up to 10 million-associated deaths. 2 CKD is a complex disease associated with a spectrum of pathological, hereditary, and sociodemographic factors. 3 There are multiple risk factors known to contribute to a decline in kidney function, including age (≥60 years), existing co-morbidities, smoking, low socioeconomic status, cardiovascular disease, a body mass index (≥30 kg/m ^2^ ), family history, and the use of certain medications. 4 5 6 7

Access to “joined up” service data from across the health system is required to improve our understanding of CKD. 8 9 10 CKD research would benefit from linked longitudinal de-identified pathology, morbidity, and mortality data that can be used by nephrology researchers to derive insights to enable better CKD patient plans and outcomes. 8 9 10 By accessing data from across the health systems, we can collect information about an individual when they present to a broad range of medical (both public and private sector) and community health services. 11 These large and complex data reservoirs provide the foundation to unlock potential within administrative-based “big data” assets. 12 13

The availability of new types of previously unlinked administrative data, provides opportunities to look at early-stage CKD patient pathways. Big data 12 13 linked assets, based on data collections used to manage, monitor, assess, and review a wide range of health conditions and services, provide unique opportunities to exploit the advanced data science and big data analytics techniques to understand factors influencing CKD disease progression. These linked data assets can provide practical insights and improve clinical understanding and decision making for CKD patients. 12 13 14

Worldwide however, a relatively small number of health systems have established and integrated a central population health database for research purposes, including countries such as Singapore, 15 Taiwan, 16 Denmark, 11 Sweden, 17 and Japan. 18 In most other health systems, missing or inconsistent data and privacy concerns with data sharing and linkage across organisations, 11 present major challenges.

While having one of the best health care systems in the world, Australia is no exception to the challenges associated with data integration within and across jurisdictions. 19 However, these challenges are magnified in Australia by a complex health care landscape which receives its funding through both public (across State, Territory and Australian government arrangements) and private pathways. 19 These distinctive reimbursement pathways often encourage siloed data storage without the option for interoperability and effective data sharing. 19 For CKD research, data challenges emerge when CKD patients undergo pathology tests at different pathology providers (typically private companies). This presents challenges when we attempt to understand the characteristics of CKD in the population through fragmented pathology pathways that are not integrated together. 20

While some government administrative health collections in Australia are routinely linked and analyzed, important health information such as general practice records and pathology data collected in the private sector 10 has not typically been available for research. 21 The underlying barriers limiting access to these datasets can be attributed to several factors, including privacy regulations, siloed health systems, and a reluctance to release personal information within and across organizational boundaries. 10 Although barriers affecting data sharing are being addressed through open data policies, more work is needed for governments to connect with the private industry to maximize information available on community needs, 22 23 while ensuring privacy concerns are recognized and mitigated.

Privacy preserving record linkage (PPRL) is a data linkage technology that allows organizations to securely link data without releasing personally identifiable information (PII), while maintaining linkage accuracy. 24 The technology has been tested and proven in a several linkage projects to support targeted early interventions, involving non-health datasets such as education, housing, and justice datasets. 22 23 PPRL has been a burgeoning research area, within numerous methodologies proposed in the academic literature. 25 It has also seen adoption in several forms across the world for real-world projects, including the phonetic-hash-based EUPID used in European cancer research. 26 Within Australia, PPRL methods utilizing Bloom filters have received adoption, including the LUMOS project connecting primary and secondary care data. 27 Bloom filter encodings have the key advantage of providing tolerance for spelling mistakes and other typographical errors, important for ensuring high linkage quality and robust analytical findings. 23 24 28 PPRL is an important technique enabling the linkage of otherwise unavailable private pathology datasets assisting to improve our understanding of the CKD and contributing to the clinical management of CKD. 10 28

## Objectives

Using privacy preserving linkage techniques, the first objective of this research study is to link data from the four largest pathology service providers in Western Australia (WA) to WA hospital, emergency, and mortality data to identify a CKD cohort for analysis. PPRL provides the main enabling methodology to create a linked repository across pathology and hospital data collections. This study aims to demonstrate the use of PPRL as a viable method allowing for collaborative analysis of big datasets across different organizations while maintaining patient privacy. In further studies, linked data will enable the mapping of the burden of CKD in WA, including the incidence, prevalence, rate of progression, and economic cost to the health system.

## Methods

### Data Collections

**Fig. 1:**
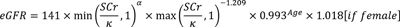
CKD-EPI equation. CKD-EPI, chronic kidney disease epidemiology collaboration.

Retrospective pathology biochemistry data was extracted from participating pathology providers (PathWest, Australian Clinical Laboratories and Clinipath Pathology) for all Western Australians (WAs) aged 18 years or older, who had a serum creatinine test in the study period (2006–2021). Each pathology provider provided biochemistry results that utilized standardized Isotope Dilution Mass Spectroscopy calibration. 29 This enables comparable reporting of serum creatinine. Using the reported serum creatinine result, estimated glomerular filtration rate (eGFR) values were re-calculated via the chronic kidney disease epidemiology collaboration equation shown in Fig. 1 . The stages of CKD are categorized according to the individual's eGFR, and take into consideration the individuals age, body size, and gender. (Western Diagnostic Pathology, also participating in this study, was not able to provide their data in time for inclusion in this manuscript).

The CKD cohort was identified from the pathology cohort, according to the following criteria, those who were ≥18 years old, with at least two eGFR results of <60 mL/min/1.73 m ^2^ performed at least 3 months apart. Additional data such as albuminuria was not available at this point for the study and has not been included for the confirmation of kidney damage. This definition will enable subsequent investigations of fluctuating trajectories of renal function.

Morbidity data for the CKD cohort was identified through the Hospital morbidity data system which captures administrative data from all public and private hospitals in WA. Mortality data was sourced from the WA Registry of Births, Deaths, and Marriages. Emergency data was extracted from the Emergency Department Data Collection which captures WA emergency department presentations. Data was extracted from these systems between 2002 and 2021.

### Data Model

**Fig. 2:**
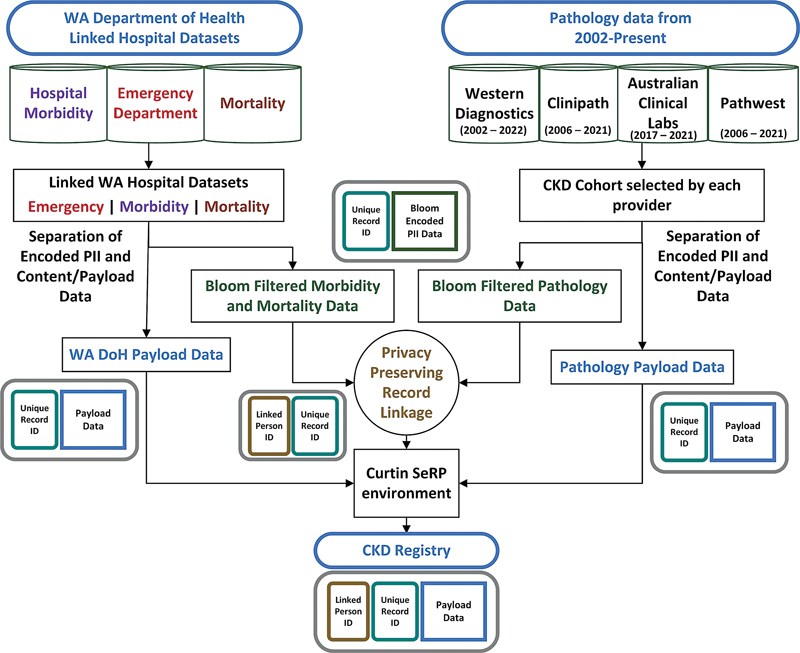
Data model and data flows.

Data linkage models were develop based on the separation principle to help reduce privacy risks. The separation principle requires data to be split into PII, which are released for linkage, and clinical content data or payload data which are provided to researchers – a unique record number connects the PII record to its clinical content record. The data model and associated data flows have been outlined in Fig. 2 . Using these data flows, which separate content data from PII during the linkage process, the risk of revealing sensitive information about individuals is significantly reduced. While our model was designed to utilize record linkage techniques based on encoded information to ensure linkers did not see any PII, a separation data model was still implemented. A common key existed between each set of personal identifiers provided to the linkage unit and its associated clinical content data provided to researchers, to allow an overarching person identifier to be attached to the research data after linkage. The registry data (pathology results, hospital admission information, etc.) accessed by researchers were not encoded in any way, this ensures easy usability by the researchers. As such, for analysts utilizing the registry, there is no difference between a dataset linked using PPRL and one linked using another linkage method.

As highlighted in Fig. 2 , PII data was encoded at source (i.e., by the pathology providers and the WA Department of Health [DoH]) before being provided to the Centre for Data Linkage at Curtin University for PPRL. Specific software to enable encoding was provided to all parties. Linkage occurred within a specifically designed ISO27001 certified environment at Curtin University. Linkage between the pathology datasets and WA health records utilized encoded personal identifiers (names, date of birth, sex, and address). The clinical content/payload data for each dataset was securely transferred and stored in SeRP at Curtin University. Examples of structured data stored in the secured environment for the CKD registry include de-identified and anonymized pathology and hospital records, including biochemistry markers such as creatinine and electrolytes, demographic factors such as postcode and WA emergency, morbidity, and mortality data.

### Data Linkage

Data linkage techniques were used to identify records belonging to each individual within and between the various data collections. PPRL involved carrying out data linkage of extracted data using encoded versions of the PII, allowing linkage to be performed without disclosing PII. To ensure consistency during the encoding process, an extraction template and password to encode the data were agreed between the data custodians before securely transferring encoded data to the linkage unit.

**Fig. 3:**
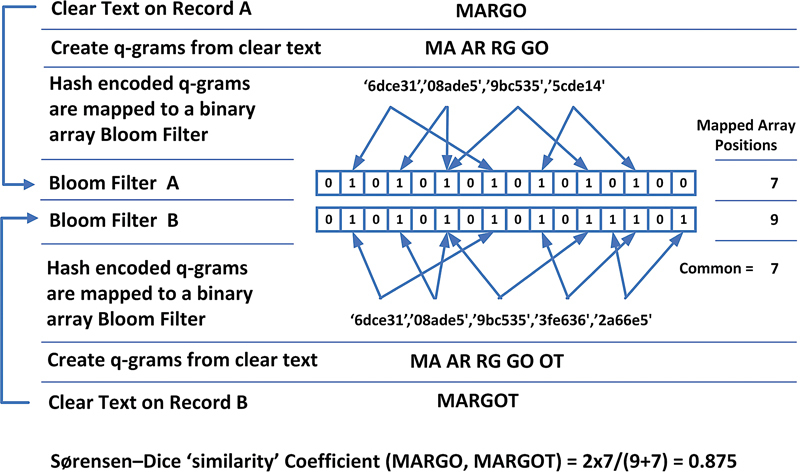
Bloom filter generation and comparison.

While several methods for PPRL have been successfully piloted, PPRL using Bloom filters has shown to offer better all-round linkage quality. 30 Consequently this method was chosen for this project. As outlined in Fig. 3 , Bloom filters are binary vectors constructed based on the letter/number combinations contained within the PII using a series of cryptographic hash functions, i.e., letter/number combinations within the PII change positions in the binary array from 0 to 1. As Bloom filters are efficient at determining whether components of the PII appear within the binary array, two bloom filters can be compared with each other based on the number of positions within the Bloom filter that match. The similarity of two Bloom filters (i.e., how many of the array positions match between two Bloom filters) allows us to decide whether records belong to the same individual or not. Further detailed technical information on Bloom filters implementation have been described by Randall et al. 31

The data was encoded for privacy preserving linkage using field-based Bloom filters; each identifier was encoded into a separate Bloom filter, with a standard probabilistic record linkage method used on these encoded identifiers. This approach has been documented in the literature previously, 31 showing higher linkage quality than other privacy preserving methods. 32

Date of birth, sex, and postcode were encoded with a single hash value, while names, address, and suburb were encoded into Bloom filters. The Bloom filter encoding used bigrams with no padding, a Bloom filter length of 512, with 30 bit positions set per bigram for names, 20 for address, and 25 for suburb. The number of bit positions set was lower for suburb and address fields as the average length of these fields was longer. Only the first 20 characters of the input fields were used in creating the encoded data. Some basic pre-processing steps occurred as part of the encoding process to standardize the data formats These included removing whitespace, converting all values to lower case, removing non-alphanumeric characters, converting to a standard format (“Street” to “ST”), and remove placeholder values (“9999” for postcode etc.).

A probabilistic linkage framework was used, utilizing the traditional Fellegi-Sunter approach, whereby individual fields of record-pairs are compared, each resulting in a score based on their agreement or disagreement, with these scores summed and accepted as the same individual if reaching a set threshold. This method had been used previously and shown to achieve high quality linkage results. All available fields (names, sex, date of birth, and address) were used in the comparison process. Fields encoded using Bloom filters were compared using the Sørensen–Dice coefficient. A default set of weights (m and u probabilities) were used; these had been developed and validated through project-based linkages previously undertaken. To reduce the number of pair comparisons, a technique called blocking was used, whereby only record-pairs that had matching values in common were compared further. The blocking strategy employed only compared records further where they had the same date of birth, or the same Soundex value of surname and first initial. These blocks have been used and validated by the linkage team in previous privacy preserving and clear-text linkages.

The datasets from the three pathology providers were deduplicated, linked together, and linked against the WA emergency, morbidity, and mortality datasets. A probabilistic linkage framework was used, utilizing the traditional Fellegi–Sunter approach, whereby individual fields of record-pairs are compared, each resulting in a score based on their agreement or disagreement, with these scores summed and accepted as the same individual if reaching a set threshold. This method had been used previously and shown to achieve high quality linkage results. 31 All available fields (names, sex, date of birth, and address) were used in the comparison process. Fields encoded using Bloom filters were compared using the Sørensen–Dice coefficient. A default set of weights (m and u probabilities) were used; these had been developed and validated through project-based linkages previously undertaken. To reduce the number of pair comparisons, a technique called blocking was used, whereby only record-pairs that had matching values in common were compared further. The blocking strategy employed only compared records further where they had the same date of birth, or the same Soundex value of Surname and first initial. These blocks have been used and validated by the linkage team in previous privacy preserving and clear-text linkages. Encoding and linkage were performed using the LinXmart software suite 33 ; linkage was performed sequentially and took approximately 10 hours to load and link all datasets to each other. Morbidity, emergency, and mortality data had been previously linked by the WA DoH's Data Linkage Branch; these links were supplied and honored within this study. The final extract from linkage included all pathology records, and any emergency, hospital, or mortality record that are linked to these pathology records. Descriptive statistics are presented outlining the links within and between these datasets.

### Ethics

The project has obtained ethics approval, (HRE2019–0303) from Curtin's Human Research Ethics Committee (HREC) for pathology datasets, and ethics approval from the DoH WA HREC for hospital datasets (RGS000000183).

## Results

Data was received from the WA DoH and three pathology providers at the time of writing, with data from the fourth pathology provider (Western Diagnostic Pathology) expected imminently. A summary description of provided datasets is shown in Table 1 . Dataset sizes differed depending on how the data was stored; for data stored in a person-based format, each dataset likely contained few duplicates; other datasets were stored in a service-based format, with each episode of care for the same individual recorded separately. The data appeared of generally high quality, with only middle name showing high levels of incompleteness.

**Table 1:** Summary of datasets received and percentage of missing information

	Department of Health			Pathology providers		
	Hospital	Emergency	Mortality	PathWest	Clinipath	ACL
*Number of records*	*19,916,472*	*18,229,362*	*270,724*	*1,727,205*	*11,352,665*	*431,169*
**Fields (% missing)**						
Given name	0.0%	0.0%	0.0%	0.1%	0.0%	0.0%
Middle name	53.0%	87.5%	23.1%	33.9%	98.6%	−
Surname	0.0%	0.0%	0.0%	0.0%	0.0%	0.0%
Sex	0.0%	0.0%	0.0%	0.0%	0.0%	5.0%
Date of birth	0.0%	0.0%	0.2%	0.0%	0.0%	0.0%
Address	0.3%	1.3%	0.3%	0.4%	0.4%	0.0%
Suburb	0.1%	1.1%	0.3%	0.3%	0.4%	0.0%
Postcode	0.0%	0.9%	1.1%	0.0%	0.4%	0.0%

A probabilistic linkage was performed to join together records within and between these six datasets. The results of this linkage identified 2,007,309 individuals (excluding those with no pathology record who are not within the study cohort). Among those identified were 1,341,444 individuals with a PathWest pathology record, 1,378,507 individuals with a Clinipath pathology record, and 79,364 people with an ACL pathology record.

**Fig. 4:**
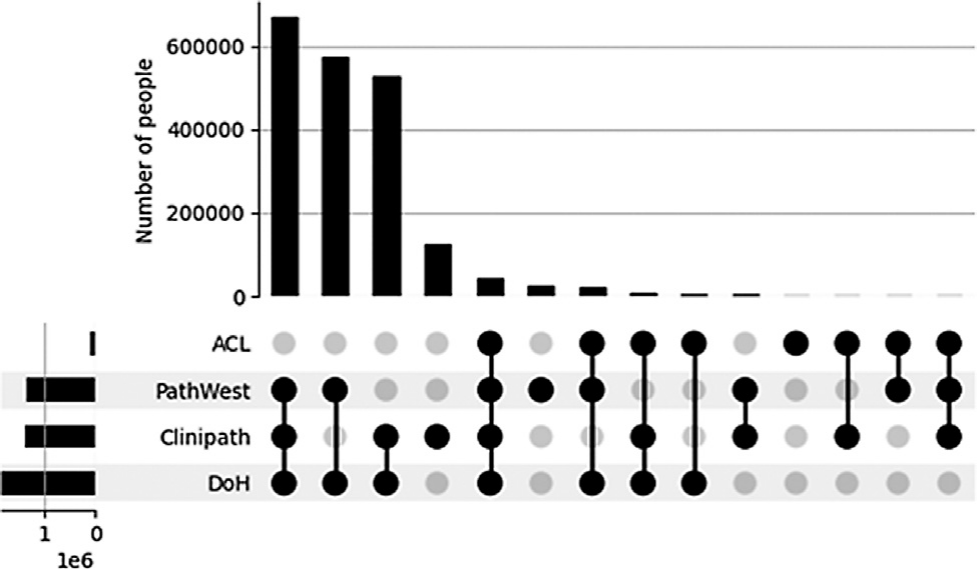
The number of individuals with records in different combinations of datasets.

Fig. 4 shows the number of individuals with records in different combinations of datasets. For instance, over 600,000 individuals in this cohort had data within the PathWest, Clinipath, and DOH collections (and no others), while just under 600,000 individuals had data within just PathWest and DOH collections. Over 55% of individuals with PathWest records had pathology records with other providers; similar results were seen for Clinipath (53% of individuals had records with other pathology providers) and ACL (91% of individuals had records with other pathology providers).

## Discussion

The main challenges to data linkage around the world are not related to the technical aspects of integrating data. They are often centered on more practical elements of data sharing, especially regarding of the release of personal information across organizations. Some barriers to data sharing are related to legislative restrictions but are more often rooted in the organization's security concerns to share data for research, where avoiding data sharing mitigates any associated privacy risk.

Hesitancy toward data sharing is being challenged through open data policies and a need for government to work with private industry to maximize information available on community needs. It is also clear that linkage systems using encryption and other advanced techniques to mask data prior to linkage will make the process of data sharing and linkage easier. Barriers can be further reduced by mitigating risks associated with the data sharing process (through careful planning, secure protocols, legal agreements, and encoding techniques).

In this study, the research team aimed to maximize the use of available health data on CKD by integrating data resources from pathology providers and the WA DoH. By removing the need to use unmasked identifiers for data linkage, PPRL systems have been adapted and scaled with sufficient quality to augment current approaches to data linkage.

The linkage results found here demonstrate the benefits of record linkage approach when utilizing data sourced from pathology providers. Over half of individuals within the cohort had pathology records from more than one provider. Consequently, sourcing and utilizing data from a single provider would provide an incomplete pathological picture for over half of our cohort. This study is the first that used privacy preserving linkage to link data obtained from these pathology providers, whereas previous studies provided datasets that were unlinked and analyzed in isolation. This study contributes to greater acceptance of data linkage techniques among pathology providers and an expected key outcome from this study will be the increased availability of pathology data for linkage for other research studies.

Through the use of privacy preserving linkage methods, we are able to satisfy custodian concerns around privacy of their patient data and join these datasets together, providing a more complete picture necessary for accurate research. By obfuscating personally identifying information before data release, privacy preserving linkage increases privacy as the personal identifiers used for linkage are no longer visible to the third party carrying out linkage and remain solely with the data custodian. These privacy preserving methods are not impenetrable, with potential attacks 34 on the encodings and their mitigations 35 documented in the literature. In discussions of privacy, it is important to consider the particular context in which the methods are being used. Our use of Bloom filter encodings involves their release to a trusted third party, which has strong information governance standards in place, and with significant legal and contractual safeguards around data transfer and use. Bloom filters are a tool used to reduce the risk of accidental or purposeful re-identification of individuals by the small number of designated users with access to the encoded data. For this purpose, they meet the required security level of data custodians who utilize them.

The results also highlight the importance of linking information from private health care providers. A significantly large percentage of individuals (47%) from Clinipath, a private pathology provider, did not have records with other pathology providers, hence demonstrate private pathology provider's central role in routine health care provision and could therefore provide greater insight into other areas of health in the community.

As all identifying information was encoded, it was not possible to directly check whether a particular pair of records belonged to the same individual, and so it was not possible to calculate quality metrics to gain an indication of the accuracy of linkage. However, the linkage method used here has been evaluated for similar linkages, achieving very high results 31 32 36 including real-world conditions, 30 with false positive rates below 1%. In addition, the linkage was designed to make use of the results of previous clear-text linkage of the Western Australia health data by the WA data linkage branch, which has been linked to a very high standard. Honoring and leveraging these previous links help improve overall linkage quality. 30 37 As such, the resulting linkage quality in this study is expected to be high.

The results presented in this paper provide only a preliminary insight into the potency of these datasets. Data from Western Diagnostic Pathology, the remaining WA pathology provider, was excluded with data arrival for this project expected imminently. Therefore, results provide an incomplete picture, and initial analysis suggests a greater level of overlap between pathology datasets once remaining data are included.

## Conclusion

Privacy preserving linkage gives confidence to data custodians through its ability to provide a secure and efficient access to large integrated datasets. It presents a useful and practical tool to enable integration and access to datasets which was otherwise not available, unlocking potential within these data resources to improve public health.

## Clinical Relevance Statement

PPRL is demonstrated as a viable method of linking pathology data between different pathology providers. Given the longitudinal nature of CKD, the linked pathology datasets enable the identification of disease which was previously impossible to identify from siloed sets of records from a single provider. PPRL also enabled linkage to hospital records that contain clinical procedural and diagnosis codes in addition to administrative demographic details, which further improve the identification of risk factors associated with CKD and the burden of disease on the health system. This information will help clinicians to prioritize early detection intervention and optimum management for patients, and improve resource allocation within health systems.

## Multiple Choice Questions

1. What is data linkage (also known as medical record linkage)?
  - A process of promoting medical research partnerships.
  - Process of identifying, matching, and merging records that correspond to the same person/entity from within and/or across several datasets.
  - Process that feeds data sandbox environments.
  - Process that offers low-cost data storage.
  **Correct Answer:** The correct answer is option b. Record linkage is the process of determining records that belong to the same person/entity within and/or between datasets.
2. What is the purpose of Privacy-Preserving Record Linkage (PPRL)?
  - To check and ensure the integrity of unit level Personally Identified Data (PII) for linkage.
  - To reduce the number of records that need to be released to a Trusted Third Party for linkage.
  - To integrate data without the need to release Personally Identified Data (PII) to a Trusted Third Party for linkage.
  - To reduce the need for Trusted Third Party organizations to link data.
  **Correct Answer:** The correct answer is option c. Privacy preserving record linkage methods allow data to be linked without the release of personally identifying information.
3. What are the benefits of PPRL?
  - Reduces reliance on large databases and data lakes.
  - Creates a data streaming channel.
  - Improves the opportunities to share data across data providers.
  - Provides off-load low-usage data availability.
  **Correct Answer:** The correct answer is option c. By reducing the privacy risk of record linkage, PPRL can improve opportunities for data to be shared and linked across providers.
